# DNA recovery from used malaria RDT to detect *Plasmodium* species and to assess *Plasmodium falciparum* genetic diversity: a pilot study in Madagascar

**DOI:** 10.1186/s12936-022-04246-y

**Published:** 2022-07-26

**Authors:** Voahangy Hanitriniaina I. Andrianaranjaka, Elisabeth Ravaoarisoa, Tovonahary A. Rakotomanga, Fanomezantsoa Ralinoro, Danielle A. Doll Rakoto, Ranjàna H. Randrianarivo, Victor Jeannoda, Arsène Ratsimbasoa

**Affiliations:** 1 Mention Biochimie Fondamentale et Appliquée, Faculté des Sciences, Université d’Antananarivo, Antananarivo, Madagascar; 2 Programme National de Lutte contre le paludisme, Ministère de la Santé Publique, Antananarivo, Madagascar; 3 Faculté de Médecine, Université de Fianarantsoa, Fianarantsoa, Madagascar; 4 Centre National d’Application de Recherche Pharmaceutique, Antananarivo, Madagascar

**Keywords:** RDT, Molecular biology, *Plasmodium falciparum*, Genetic diversity, Multiplicity of infection, Madagascar

## Abstract

**Background:**

Rapid diagnostic tests (RDT) are widely used for malaria diagnosis in Madagascar, where *Plasmodium falciparum* is the predominant species. Molecular diagnosis is essential for malaria surveillance, but requires additional blood samples for DNA extraction. Used RDTs is an attractive alternative that can be used as a source of DNA. *Plasmodium falciparum* genetic diversity and multiplicity of infection, usually determined by the genotyping of polymorphic regions of merozoite surface proteins 1 and 2 genes (*msp1*, *msp2*), and the repeated region RII of the *glutamate-rich protein gene (glurp)* have been associated with malaria transmission levels and subsequently with the impact of the deployed control strategies.

Thus, the study aims to use RDT as DNA source to detect *Plasmodium* species, to characterize *Plasmodium falciparum* genetic diversity and determine the multiplicity of infection.

**Methods:**

A pilot study was conducted in two sites with different epidemiological patterns: Ankazomborona (low transmission area) and Matanga (high transmission area). On May 2018, used RDT (SD BIOLINE Malaria Ag P.f/Pan, 05FK63) were collected as DNA source. *Plasmodium* DNA was extracted by simple elution with nuclease free water. Nested-PCR were performed to confirm *Plasmodium* species and to analyse *P. falciparum msp1*, *msp2* and *glurp* genes polymorphisms.

**Results:**

Amongst the 170 obtained samples (N = 74 from Ankazomborona and N = 96 from Matanga), *Plasmodium* positivity rate was 23.5% (40/170) [95% CI 17.5–30.8%] by nested-PCR with 92.2% (37/40) positive to *P. falciparum*, 5% (2/40) to *Plasmodium vivax* and 2.5% (1/40) to *P. falciparum/P. vivax* mixed infection. Results showed high polymorphisms in *P. falciparum msp1*, *msp2* and *glurp* genes. Multiple infection rate was 28.6% [95% CI 12.2–52.3%]. The mean of MOI was 1.79 ± 0.74.

**Conclusion:**

This pilot study highlighted that malaria diagnosis and molecular analysis are possible by using used malaria RDT. A large-scale study needs to be conducted to assess more comprehensively malaria parasites transmission levels and provide new data for guiding the implementation of local strategies for malaria control and elimination.

*Trial registration* Retrospectively registered

## Background

Despite the deployment of considerable efforts and various strategies to control and eliminate malaria, this disease remains one of the main cause of morbidity and mortality worldwide [1]. In 2020, 241 million cases were reported leading to 627,000 deaths [2]. Sub-Saharan Africa, including Madagascar, still remains the most affected region [2]. Among the five *Plasmodium* species infecting humans, *Plasmodium falciparum* is the most prevalent malaria species in sub-Saharan Africa and the most virulent species leading severe malaria [3].

Rapid Diagnostic Tests for malaria (RDT) are commonly used for malaria diagnosis in field by community health workers to promptly manage malaria cases. Malaria molecular diagnosis are essential for epidemiological surveillance, but requires to collect additional blood samples for DNA extraction. Used RDTs is an attractive alternative that can be used as a source of DNA [4–6].

*Plasmodium falciparum* genotyping remains an important tool for studying types and numbers of parasite clones present in an infection. Currently, this approach is mainly used to investigate the genetic diversity of parasites in human infections and subsequently estimate the malaria transmission intensity [1, 7–10]. Moreover, numerous studies have demonstrated an association between high multiplicity of infection (MOI) and malaria severity, especially in high malaria transmission areas [11]. Usually, *P. falciparum* genetic diversity is determined by the genotyping of the polymorphic regions of the block 2 of merozoite surface protein-1 (*msp1*), block 3 of merozoite surface protein-2 (*msp2*) and the RII repeated region of the glutamic rich protein genes (*glurp*) [12–15].

In Madagascar, four to eight malaria ecozones are described according to their different epidemiological profile that range from low to high transmission [16, 17]. Regular molecular and epidemiological monitoring over time and space of the genetic diversity of *P. falciparum* populations in association with malaria phenotypes (uncomplicated malaria, severe malaria and asymptomatic malaria cases) is crucial to evaluate the impact of malaria control interventions and to guide the deployment of local tailored strategies for elimination [1, 11, 18, 19].

Hence, this study aims to valorize blood samples collected onto RDT as DNA source for detecting *Plasmodium* infections, assessing the genetic diversity of *P. falciparum* populations and estimating the MOI in *P. falciparum* isolates collected from symptomatic patients seen in health centres in two sites with different epidemiological patterns: Ankazomborona, located in a low transmission area and Matanga in a high transmission area.

## Methods

### Study sites and sample collection

**Fig. 1:**
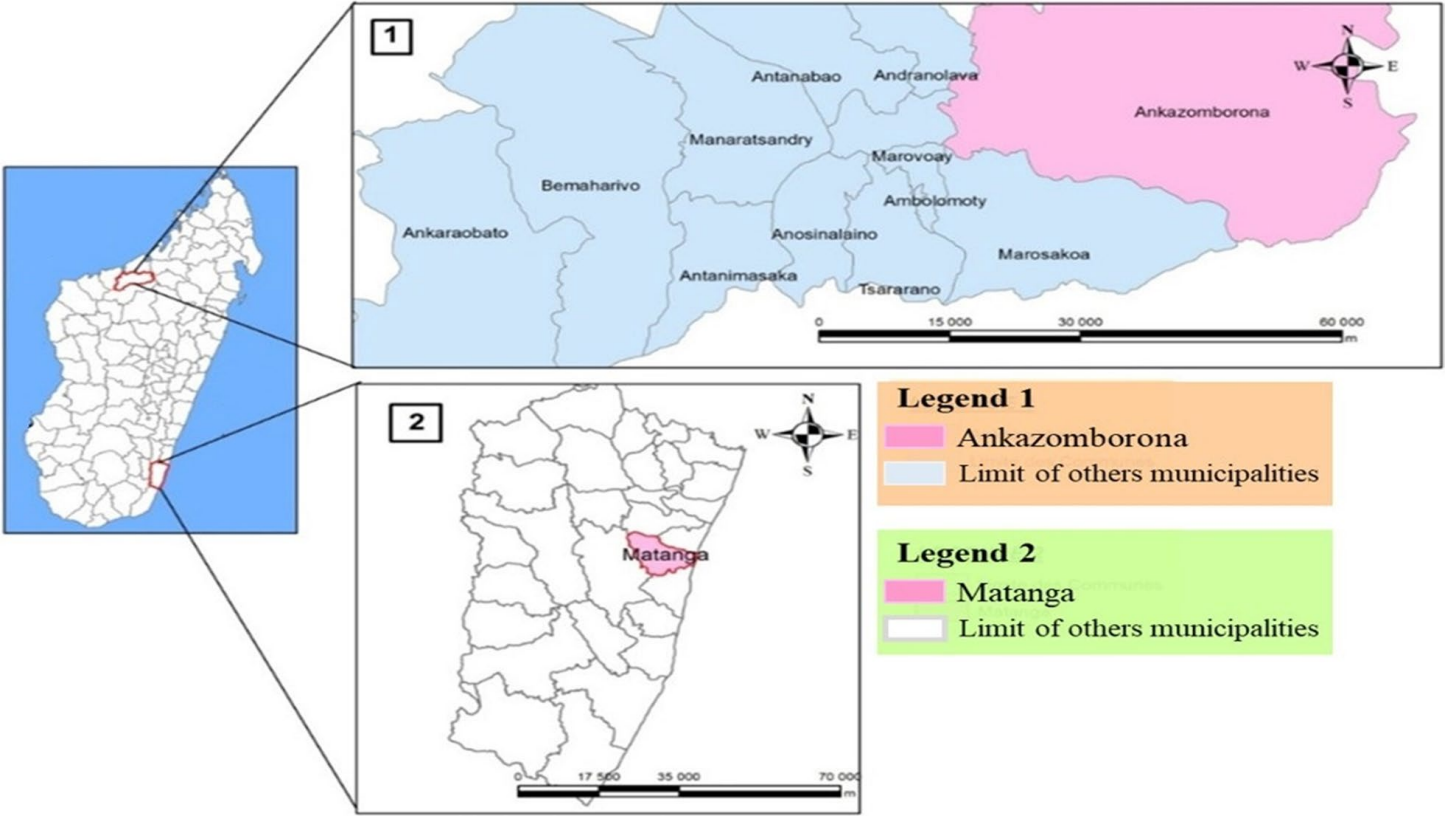
Geographical situation of Ankazomborona and Matanga (Source: BD 500 and FTM)

The study was conducted on May 2018 in two sites: in Ankazomborona, district region of Marovoay, located on the west coast of Madagascar (16° 07′ 00″ S and 46° 45′ 00″ E) and in Matanga, district region of Vangaindrano, located on the east coast of Madagascar (23° 31′ 00″ S and 47° 33′ 00″ E) (Fig. 1). Ankazomborona is in the tropical stratum area with a seasonal, endemic and low transmission defined as 10–50 cases per 1000 population per year. While, Matanga is in equatorial stratum area with a perennial endemic and high transmission defined as more than 100 cases per 1000 population per year [20]. Malaria prevalence by microscopy among children 6–59 months of age was 9.0 and 8.8% respectively, in the zones encompassing Ankazomborona and Matanga in 2016 [21]. *Plasmodium*-positive RDT (SD BIOLINE Malaria Ag P.f/Pan, 05FK63) were collected. They were obtained from children aged 6 months to 15 years suffering to uncomplicated malaria. These RDT containing blood samples were conserved at room temperature before DNA extraction.

### DNA extraction

**Fig. 2:**
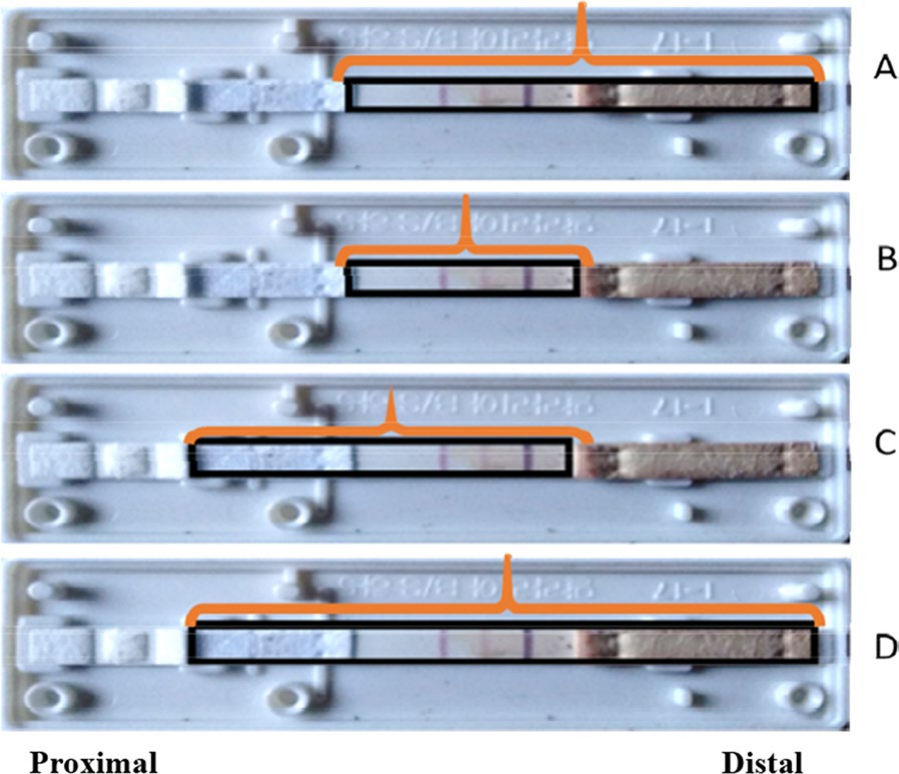
Fragment sampling of RDT strip. **A** Distal part, **B** central part, **C** proximal part, **D** all parts

To improve the protocol aiming at extracting parasite DNA from RDT, EDTA tubes containing whole blood infected by *P. falciparum* and *P. vivax* were used. Blood smears were read to identify *Plasmodium* species and estimate the parasite density (parasites/µL). Blood samples were then diluted with non-infected blood to obtain aliquots containing parasitaemia ranging from 1500 to 5 parasites/µL. Four RDT were tested for each dilution. The cassettes of RDT were opened laterally and the strips were taken out and cut for DNA extraction. For each parasite densities, four different parts of the strip were used to estimate the best yield of DNA extract: (A) distal part, (B) central part, (C) proximal part and (D) all parts (Fig. 2).

Two methods of DNA extraction were applied: the Instagene Matrix^©^ method (BioRad™), according to the manufacturer’s instructions and a simple elution method in water as previously described [4].

### *Plasmodium falciparum* and *Plasmodium vivax* detection by nested-PCR

Nested-PCR [22] was performed to detect *P. falciparum* and *P. vivax* DNA and estimate which DNA extraction method and which part of RDT strip provide the more reliable results. Nested-PCR assays showed that all parts of RDT strip and simple elution method in water were the best approaches (see “Results” section). These methods were then selected for all further experiments.

### *Plasmodium falciparum msp1*, *msp2* and *glurp* genotypin*g*

The polymorphic region of *msp1*, *msp2* and *glurp* were genotyped using nested-PCR. Primers targeting the block 2 region of *msp1*, the block 3 region of *msp2*, and the RII repeated region of *glurp* were used for primary PCR (Table 1). All PCR reactions were carried out in a total volume of 25 μL, containing 200 nM dNTP mix, 2 mM MgCl_2_, 200 nM each of forward and reverse primers for both *msp1*, *msp2* and *glurp*, 0.5 U of Taq DNA Polymerase (Bioline) and 3 μL of extracted DNA, used as template. PCR amplification of *msp1*, *msp2* and *glurp* genes comprised an initial step of 94 °C for 5 min followed by 30 cycles of 94 °C for 30 s, 55 °C for 30 s, 72 °C for 1 min 30 s, and a final extension of 72 °C for 5 min.

**Table 1:** Primary and secondary PCR primers

Gene	Allelic type	PCR round	Primer sequence (5′–3′)	Fragment size (bp)
*Msp1*		Primary PCR	CACATGAAAGTTATCAAGAACTTGTC	633
			GTACCGCTAATTCATATTCTATTGCTAG	
	MAD20	Nested PCR	GAACAAGTSGAACAGCTGTTA	120–250
			TGAATTATCTGAAGGATTTGTACGCCT	
	K1	Nested PCR	GAAATTACTACAAAAGGTGCCAAGTG	160–300
			AGATGAAGTATTTGAACGAGCTAAAGT	
	RO33	Nested PCR	GCAAATACTCAAGTTGTTGCAAAGC	100–160
			AGGATTTGCAGCAYCCTGGAGATCT	
*Msp2*		Primary PCR	ATGAAGGTAATTAAAACATTGTCTATTAAT	811
			ATATGGCAAAAGATAAAACAAGTGTTGCTG	
	3D7	Nested PCR	GCAGAAAGTAAKGCCTYTCTACTGGTGCT	150–350
			AGATGAAGTATTTGAACGAGGTAAAGTG	
	FC27	Nested PCR	GCAAATGAAGGTTCTAATACTAATAG	300–600
			GCTTTGGGTCCTTCTTCAGTTGATTC	
*Glurp*		Primary PCR	ATG AAT TYG AAG ATG TTC ACA CTG AAC	1200
			ATG AAT TYG AAG ATG TTC ACA CTG AAC	
		Nested PCR	CTG AAC CAA ATCA AAA TAA CG	600–1000
			TTC TTC TGG TTT TAT AGT TTC	

For the nested-PCR, specific primers to allelic families of *msp1* (MAD20, K1, and RO33), and *msp2* (3D7 and FC27) were used. For *glurp* amplification, inner primers were used to amplify generated amplicons (Table 1). This secondary reaction contained the same reagents as the primary reaction except primers (Table 1) and 2 μL of the primary PCR product was used as DNA template. The cycling profile for the secondary PCR was similar to the primary PCR for *glurp* while for *msp1* and *msp2* the annealing temperature was increased from 55 to 60 °C. DNA from non-infected blood and from reference *P. falciparum* strains (3D7, Dd2 and 7G8) were included in each set of PCR reactions as a negative and positive controls.

Eight microlitres of nested PCR products were loaded on 2% agarose gel stained with ethidium bromide and separated by electrophoresis for an average of 60 min at 120 V. After electrophoresis, the gels were visualized under UV trans-illumination using Image Lab gel doc system and then analysed to estimate the bands sizes. PCR products size were estimated by Image Lab software using 100 bp DNA ladder marker.

Polyclonal infection was defined by the presence of more than one allele for a given gene [23, 24]. Multiplicity of infection (MOI) was defined by the number of genotypes per infection [25].

## Results

### Optimization of DNA extraction

**Fig. 3:**
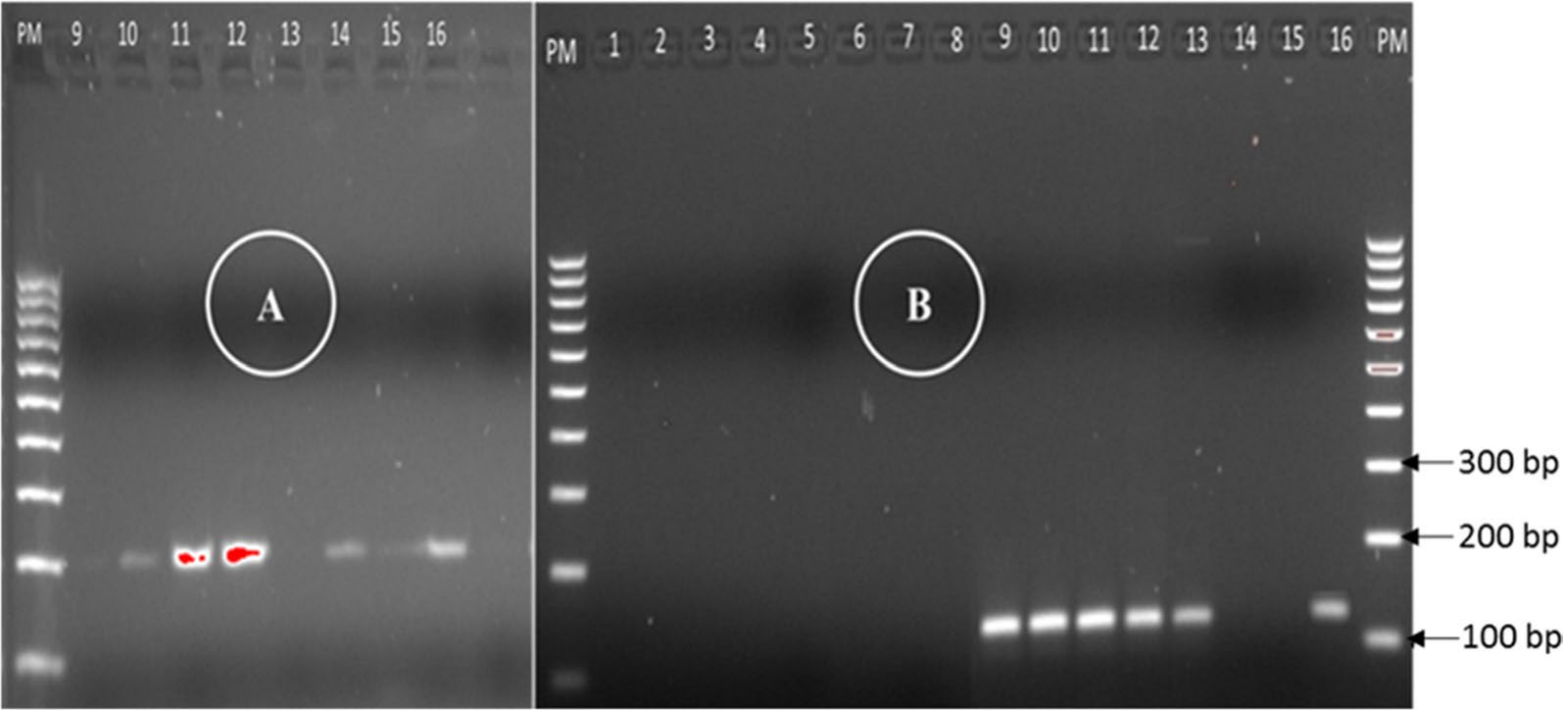
PCR product from DNA extracted by the two methods: Instagena matrix (1 to 8) and simple elution in water (9 to 16). Expected size 206 bp for *P. falciparum* (**A**) and 120 bp for *P. vivax* (**B**), PM: 100 bp DNA ladder marker, 1–16: samples

Out of 16 RDT samples (8 tested with *P. falciparum* infected bloods and 8 by *P. vivax* infected bloods), DNA extracted from all parts of RDT strip and simple elution method in water gave successful results by PCR amplification (Fig. 3).

### *Plasmodium falciparum* and *Plasmodium vivax* infections by nested PCR

Out of 170 samples of used RDT (74 from Ankazomborona and 96 from Matanga) analysed, nested-PCR allowed to detect 40 positives samples (23.5%, 95% CI 17.5–30.8%) of which 37 were *P. falciparum* (92.5%, 95% CI 78.5–98.0%), 2 *P. vivax* (5%, 95% CI 0.9–18.2%) and 1 *P. falciparum*/*P. vivax* mixed infection (2.5%, 95% CI 0.1–14.7%) (Table 2).

**Table 2:** Positivity of P. falciparum and P. vivax by nested PCR

	Ankazomborona (n = 74)		Matanga (n = 96)		Total (N = 170)	
	n	%	n	%	n	%
*P. falciparum*	16	21.6	21	21.9	37	21.8
*P. vivax*	2	2.7			2	1.2
*P. falciparum* + *P. vivax*	1	1.4			1	0.6
Negative	55	74.3	75	76.5	130	76.5

### Frequency of *msp1* and *msp2* allelic families

Out of all 38 *P. falciparum* positive samples, 21 were successfully amplified for *msp1* (55.3%, 95% CI 38.5–71.0%) and 9 for *msp2* (23.7%, 95% CI 12.0–40.6%). For *msp1*, MAD20 was the most frequent allelic family and detected in 52.4% (95% CI 30.3–73.6%) of the samples, followed by RO33 (47.6%; 95% CI 26.4–69.7%) and K1 (28.6%; 95% CI 12.2–52.3%). A total of 28.6% of the positive samples for *msp1* contained polyclonal infection with MAD20/K1, MAD20/R033 and K1/R033. For *msp2*, all positive samples (9/9) belonged to the FC27 allelic family and were classified as monoclonal infection. None of the 3D7 allelic family was detected.

### Genetic diversity, allelic frequency and multiplicity of infection

Generated alleles were classified according to their fragment sizes for *msp1*, *msp2* and *glurp*. Allelic frequency for an allele was defined as being its proportion compared to the total number of all detected alleles in the whole isolates. *Plasmodium falciparum* isolates in both sites were highly polymorphic, since almost alleles detected were once. For *msp1* gene, 14 different alleles were observed: 4 alleles for MAD20 (size range 150–250 bp), 1 allele for RO33 (size range 120–150 bp) and for 6 alleles for K1 (size range 140–300 bp) (Table 3). Only one sample from Matanga presented polyclonal infection for MAD20 allelic family (MAD20-D) (Table 3).

**Table 3:** Alleles distribution for Msp1

Allele	Fragment size (bp)	Ankazomborona n (%)	Matanga n (%)	Total N (%)
MAD20 allelic family				
MAD20-A	150	1 (9)	0 (0)	1 (9)
MAD20-B	200	5 (46)	2 (18)	7 (64)
MAD20-C	250	0 (0)	2 (18)	2 (18)
MAD20-D	200 + 250	0 (0)	1 (9)	1 (9)
Total for MAD20		6 (55)	5 (45)	11 (100)
RO33 allelic family				
RO33	120–150	5 (50)	5 (50)	10 (100)
Total for RO33		5 (50)	5 (50)	10 (100)
K1 allelic family				
K1-A	140	0 (0)	1 (≈ 17)	1 (≈ 17)
K1-B	160	1 (≈ 17)	0 (0)	1 (≈ 17)
K1-C	180	0 (0)	1 (≈ 17)	1 (≈ 17)
K1-D	220	1 (≈ 17)	0 (0)	1 (≈ 17)
K1-E	240	0 (0)	1 (≈ 17)	1 (≈ 17)
K1-F	300	0 (0)	1 (≈ 17)	1 (≈ 17)
Total for K1		2 (33.3)	4 (67.7)	6 (100)

**Fig. 4:**
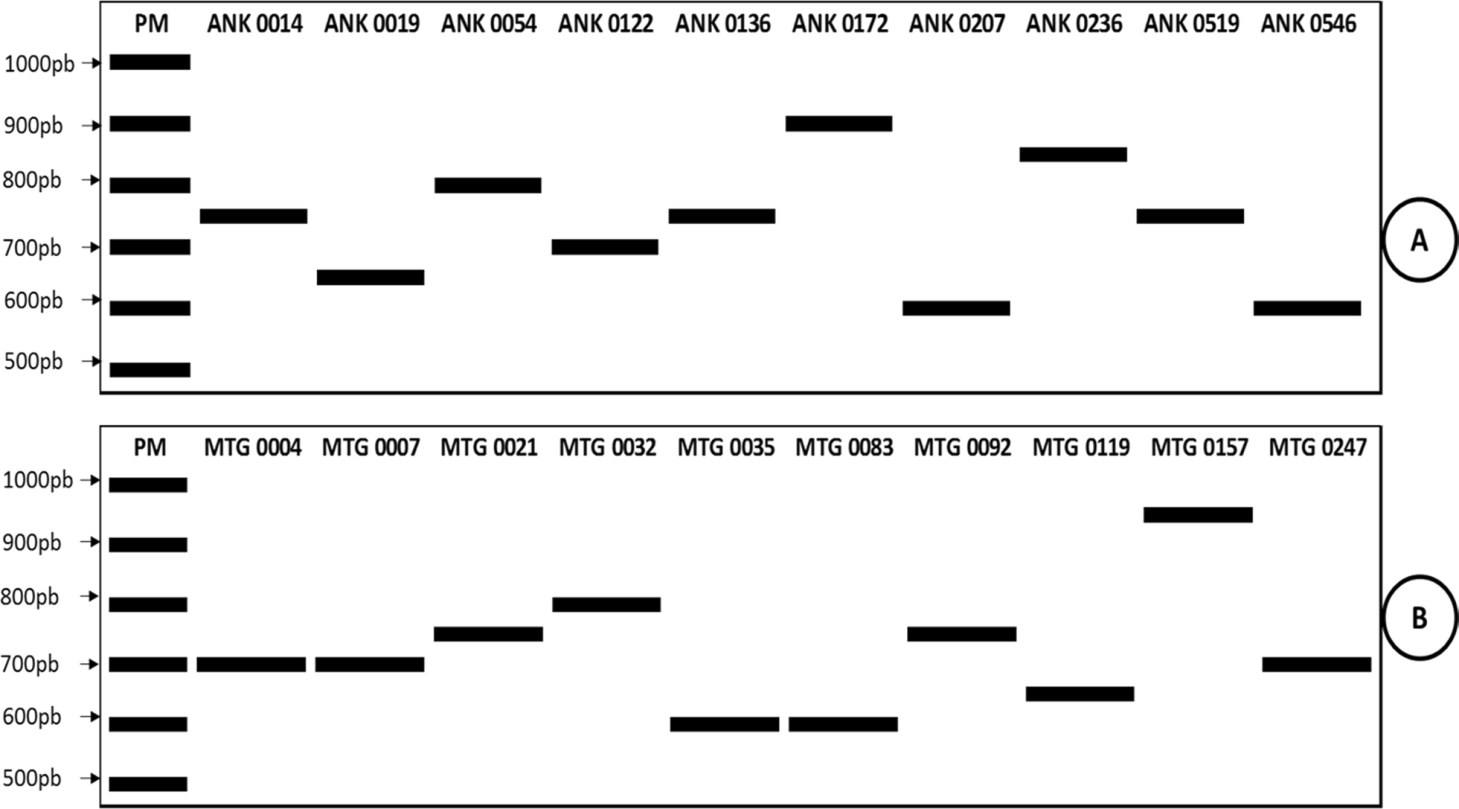
Polymorphism of *P. falciparum* *glurp* gene

For *msp2* gene, only the FC27 allelic family was observed but the genetic diversity was high: 6 different alleles were detected among the 9 positive samples (Table 4). For *glurp* gene, out of the 38 *P. falciparum* positive samples, 20 (52.6%; 95% CI 36.0–68.7%) were successfully amplified. As for *msp1* and *msp2* genes, a high proportion of different alleles (N = 8) was observed (Fig. 4A, B, Table 5).

**Table 4:** Allele distribution for Msp2 (FC27 allelic family)

Allele	Fragment size (bp)	Ankazomborona n (%)	Matanga n (%)	Total N (%)
FC27-A	420	1 (11)	0 (0)	1 (11)
FC27-B	440	1 (11)	2 (22)	3 (33)
FC27-C	460	2 (22)	0 (0)	2 (22)
FC27-D	500	1 (11)	0 (0)	1 (11)
FC27-E	520	0 (0)	1 (11)	1 (11)
FC27-F	540	0 (0)	1 (11)	1 (11)
Total		5 (55.6)	4 (44.4)	9 (100)

The mean value of MOI was 1.79 ± 0.74, similar in both sites (1.79 ± 0.80 in Ankazomborona and 1.79 ± 0.70 in Matanga). No significant difference in MOI was also observed between *msp1* (1.33 ± 0.48), *msp2* (1.0 ± 0) and *glurp* (1.0 ± 0) genes.

**Table 5:** Allele distribution for Glurp

Allele	Fragment size (bp)	Ankazomborona n (%)	Matanga n (%)	Total n (%)
Glurp-A	600	2 (10)	2 (10)	4 (20)
Glurp-B	650	1 (5)	1 (5)	2 (10)
Glurp-C	700	1 (5)	3 (15)	4 (20)
Glurp-D	750	3 (15)	2 (10)	5 (25)
Glurp-E	800	1 (5)	1 (5)	2 (10)
Glurp-F	850	1 (5)	0 (0)	1 (5)
Glurp-G	900	1 (5)	0 (0)	1 (5)
Glurp-H	950	0 (0)	1 (5)	1 (5)
Total		10 (50)	10 (50)	20 (100)

## Discussion

### Used RDT as source of DNA for molecular analysis

This preliminary study, focused on *Plasmodium* species detection and *msp*1, *msp*2 and *glurp* genotyping, was performed to evaluate the feasibility of using used RDT, collected from public health facilities in Madagascar, as source of DNA for molecular analysis. Previous findings revealed that used RDTs provide a valuable source of parasite DNA, which can be used for molecular analysis [4–6, 26, 27]. Two DNA extraction methods, Instagene Matrix^©^ method (BioRad™) and simple water elution method, were tested in the present study. Compared methods for DNA extraction showed that simple water elution method gave higher successful result. Thus, this method was adopted in the study. One hundred seventy used RDT samples were analysed. The proportion of *Plasmodium* infection was 23.5% (95% CI 17.5–30.8%) including 92.2% *P. falciparum* positive, 5% *P. vivax* positive and 2.5% *P. falciparum/P. vivax* mixed positive samples. Although this low proportion, this study highlighted the opportunity using used RDT for malaria diagnosis and molecular analysis [4]. Furthermore, studies confirmed that they also can be useful for molecular markers surveillance and whole genome sequencing [28, 29]. The main advantage is that no other consumable, nor reagent for blood sampling are needed and RDT are widely available in public health facilities since 2008 in Madagascar [30]. Nevertheless, the lack of DNA quality and quantity analysis constitute one of the limitations for the present study. Furthermore, the parasitaemia for each samples are not available and the laboratory analysis were carried out 8 months after the collection of used RDT that were stored two months in field. Thus, the storage of used RDT in the field conditions might have likely affected the DNA integrity. DNA extraction method may should be improved for increasing the yield of parasite DNA [5]. Yet, several parameters such as parasitaemia and the impact of storage duration and conditions of used RDT on the quality of the DNA extract should be considered. Besides, in order to increase the power of the study, conducting analysis with large samples is needed.

### Genetic diversity and multiplicity of *Plasmodium falciparum* infection

Out of 38 confirmed *P. falciparum* samples, 21 were successfully amplified for *msp1* (55.3%, 95% CI 38.5–71.0%), 9 (23.7%, 95% CI 12.0–40.6%) for *msp2* and 20 (52.6%, 95% CI 36.0–68.7%) for *glurp* genes. Compared to previous studies, this amplification rate was low [23, 31]. The small amount of blood deposited on RDT, 5 µL according manufacturer’s instructions [32, 33], might have affected extract DNA quantity. Moreover, targeting genes are in a single copy which could reduce amplification rate especially in low DNA concentration cases [34–36].

For *msp1*, the most frequent allelic family was MAD20 (52.4%), followed by RO33 (47.6%) and K1 (28.6%), similar to those found in Equatorial Guinea, Myanmar and Senegal [24, 37, 38], but in contrast to those observed in Congo DR and Yemen where the K1 allelic family predominate about 40 to 60% [39, 40].

Due to the low amplification rate and the limited samples, only the FC27 allelic family was detected for the *msp2*, concordant with data observed by Rakotomanjaka in the same locality (Matanga) in 2016 [41]. This is not the case in all localities in Madagascar, since several studies using DNA sequencing method, reported higher frequency of 3D7 allelic (up to 48%) such as in Andapa, Mahasolo and Saharevo [42, 43] [44]. Whereas, the study was carried out during the peak of malaria transmission [45].

Analysis based on the PCR product fragments showed 4 distinct alleles for MAD 20 allelic family (11 samples), 1 allele for RO33 (10 samples) and 6 alleles for K1 (6 samples). The K1 allelic family was found at low frequency (28.6%) compared to MAD20 and RO33, but this allelic family was more polymorphic (6 distinct alleles for 6 samples). For FC27 allelic family of *msp2*, out of the 9 positive samples, 6 distinct alleles were detected. Regarding to *glurp* gene, 8 distinct alleles were observed in the 20 positive samples. Polymorphism analysis showed high genetic diversity of *P. falciparum* populations in Ankazomborona and Matanga. Majority of alleles detected during in this study were single with a high polymorphism level. No significant difference of polymorphism level was detected in two study sites. Parasite genetic diversity is one of key elements to consider for strategies implementation to fight against malaria [40]. It has an impact on parasite transmission and control strategies, and it varies according to transmission in endemic areas [7–10]. According to the results, it seems, transmission intensity is similar in Ankazomborona and in Matanga. However, parasites transmission and genetic diversity could be higher during the peak of malaria transmission and may reflect the dynamics of parasites transmission [23, 25]. Hence, a study on large samples is crucial before giving final conclusion.

This study showed 28.6% multiple infection for *P. falciparum *msp1 gene not for *msp2* and *glurp*. These results indicate that polyclonal infection frequency is low compared to those found in other countries [46, 47]. The MOI average value was 1.79 ± 0.74, a lower value than in other northwestern Ethiopia (2.6), in Republic of Congo (2.64), and in Equatorial Guinea (5.5) [25, 37, 40]. MOI for Ankazomborona and Matanga did not differ significantly. Thus, hypothesis concerning the link between the high level of MOI and malaria severity, particularly in areas with high transmission rate [11], is not verified during this study due to the limited number of samples. Despite this, the present study revealed that *P. falciparum* populations circulating in Madagascar showed a high level of genetic polymorphism. That high polymorphism reflects transmission intensity. Consequently, it would be interesting to increase samples number in order to continue genetic diversity study over time and space for carrying out a cartographic study about *Plasmodium* allelic families’ distribution in Madagascar.

## Conclusion

The present study highlighted the possibility of performing malaria diagnosis and molecular genetic diversity analysis of *Plasmodium* using blood samples collected on RDT as DNA source. Preliminary data from this study showed high genetic diversity of *P. falciparum* populations in Ankazomborona and in Matanga but multiple infection rate was low in the both study sites.

This study provides fundamental information on *P. falciparum* genetic diversity and allow to update available data. The results of this study can be used as a baseline information for future studies on parasite transmission dynamics and to evaluate effectiveness malaria prevention and control strategies.

## Data Availability

The data are available from the National Malaria Control Programme of Madagascar.
